# Evaluation of the associations of pulmonary vein vertical diameter, cardiothoracic ratio, and atrial fibrillation combined hiatal hernia

**DOI:** 10.1097/MD.0000000000039583

**Published:** 2024-09-13

**Authors:** Bowen Xu, Xueshan Zhang, Wei Qian, Ran Zhou, Tao Chen, Yanfeng Ma, Hongping Chen

**Affiliations:** a Department of Cardiology, The Affiliated Hospital of Xuzhou Medical University, Xuzhou, Jiangsu Province, China; b Medical Department of Qingdao University, Qingdao, Shandong Province, China; c Medical School, Soochow University, Suzhou, Jiangsu Province, China.

**Keywords:** atrial fibrillation, cardiothoracic ratio, hiatal hernia, pulmonary vein diameter

## Abstract

Recent studies have suggested that there may be a relationship between hiatal hernia (HH) and atrial fibrillation (AF), but the specific mechanism is unclear. The aim of this study was to explore the clinical characteristics associated with HH and AF and to identify the potential relationship between the 2 diseases. The study comprised 180 patients with HH, of which 54 had AF. Every patient had chest computed tomography to quantify the cardiothoracic ratio, HH volume, thoracic cavity volume, and diameters of the pulmonary veins. The clinical data of all patients was acquired through an electronic medical record system. Patients who experienced AF had a noticeably smaller total pulmonary vein vertical diameter (TPVVD) and a higher cardiothoracic ratio compared to those who only had HH. Logistic multivariate regression study demonstrated a significant association between TPVVD, cardiothoracic ratio, and AF in individuals with HH. This study established a correlation between TPVVD, cardiothoracic ratio, and HH in conjunction with AF. Patient with HH who had a thinner TPVVD and a bigger cardiothoracic ratio were found to have a greater likelihood of suffering from AF.

## 1. Introduction

Atrial fibrillation (AF) is the most common serious arrhythmia.^[1]^ AF is mainly found in older individuals and those with lifestyle-related conditions such as high blood pressure, diabetes mellitus, and obesity.^[2]^ Based on the risk factors of AF, AF is roughly divided into “wear-and-tear” AF (that is, induced by environmental factors), congenital AF, and genetic AF. Risk factors for induced AF often include age and Western dietary and lifestyle risk factors such as hypertension, diabetes mellitus, obesity, coronary artery diseases, and various other conditions, including chronic kidney disease and inflammatory diseases.^[3,4]^

The pathogenesis of AF is still unclear. There is frequently more than 1 mechanism involved in the occurrence of AF. It is commonly accepted that AF results from a combination of mechanisms due to research on triggering, reentrant, focal ectopic activity, electrical remodeling, structural remodeling, and other potential mechanisms.^[4,5]^ Furthermore, other mechanisms that are involved include oxidative stress, genetics, inflammation, and the autonomic nervous system.^[6,7]^

A hiatal hernia (HH) is a medical condition where an organ within the abdomen, usually the stomach, bulges into the chest cavity through an opening in the diaphragm called the esophageal hiatus, causing symptoms. HH is such a common endoscopic finding (reported prevalence 20%) that it is, by definition, a normal variant and not a disease.^[8]^ Nevertheless, the impact of HH on the heart is still uncertain. Recent research indicates a potential association between HH and AF. Roy et al found that HH is linked to a greater occurrence of AF in both male and female individuals of all age groups, particularly in younger patients.^[9]^ A study discovered that AF was more prevalent in individuals with small HH, defined as a size of 2 cm or less, but not in those with large HH.^[10]^

Prior research has indicated a potential connection between HH and the initiation of AF.^[11]^ Conversely, it has also been discovered that Nissen fundoplication has been associated with a reduction in the incidence of AF in previous cases,^[12–14]^ but the exact mechanism remains unclear. The purpose of this study was to characterize patients with HH and AF from a clinical standpoint to better understand the underlying relationship between HH and AF for advising treatment decisions for clinicians.

## 2. Patients and methods

Ethics Committee approval was not required for this retrospective study. The waiver of ethical approval was granted by the Institutional Review Board of The Affiliated Hospital of Xuzhou Medical University (AF-IRB-042-01). The data were anonymized, and the requirement for informed consent was waived. All methods were performed in accordance with relevant guidelines and regulations.

### 2.1. Patient selection and data collection

This study reviewed 2710 patients diagnosed with HH via computed tomography (CT) or gastroscopy between January 2017 and December 2021. AF is diagnosed by electrocardiogram. Electrocardiographic characteristics include (1) irregular R-R intervals (when atrioventricular conduction is present), (2) absence of distinct P waves, and (3) irregular atrial activity also known as fibrillatory waves. In total, 147 patients (5.4%) were diagnosed with both HH and AF. Cachexia, hyperthyroidism, electrolyte abnormalities, moderate to severe heart failure, myocardial infarction, thoracic deformity, and rheumatic heart disease were removed from the study, leaving 54 participants with complete data. A total of 126 HH patients were selected as control group using the method of propensity score matching. Hypertension was defined as a systolic blood pressure ≥ 140 mm Hg and diastolic blood pressure ≥ 90 mm Hg.

### 2.2. CT imaging protocol

**Figure 1.**
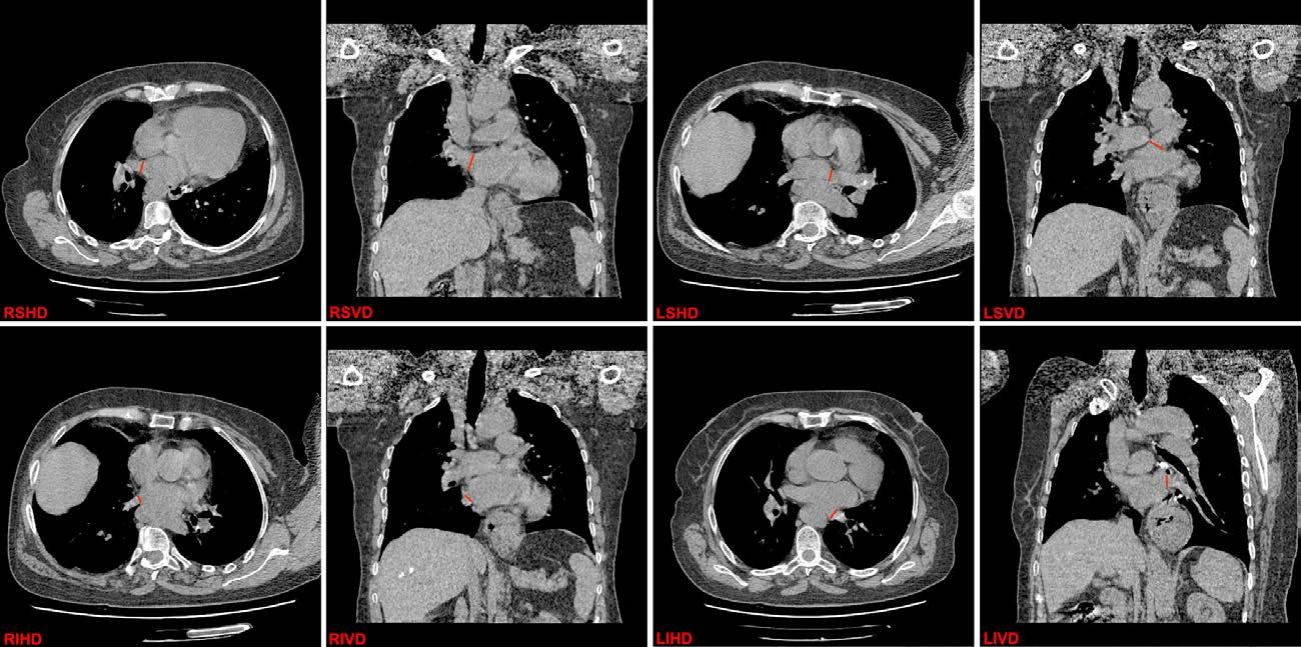
Measurement of pulmonary vein diameter.

All participants underwent CT using a Somatom Sensation 64 scanner (Siemens, Germany). The CT parameters were as follows: gantry rotation time, 330 ms; tube voltage, 120 kW and 250 mA; and detector collimation, 0.6 mm. All pulmonary vein parameters (left superior, left inferior, right superior, and right inferior pulmonary vein vertical and horizontal diameters (left superior vertical diameter, left superior horizontal diameter, left inferior vertical diameter, left inferior horizontal diameter, right superior vertical diameter, right superior horizontal diameter, right inferior vertical diameter, right inferior horizontal diameter)) and cardiothoracic ratio were measured using Medcare software (Medcare, China). Pulmonary vein diameter was measured according to previous study.^[15]^ HH and thoracic volumes were measured using 3D Slicer image computing platform (www.slicer.org). The threshold was set to 10 and 100 Hounsfield units to distinguish other structures from the pulmonary veins in the study area. The total pulmonary vein vertical diameter (TPVVD) was calculated by summing the vertical diameters of each pulmonary vein (Fig. 1).

### 2.3. Statistical analysis

MedCalc (MedCalc Software Ltd., Belgium) was used to analyze the data. For continuous variables, the data are presented as mean and standard deviation. For ordinal variables, the data are shown as medians (ranges), frequencies, and percentages. Continuous variables were analyzed using unpaired *t* tests and the Mann–Whitney *U* test (in case of non-normality); dichotomous variables were analyzed using Pearson 2 or Fisher exact test. Statistical significance was set at *P* < .05. Age, gender, presence of hypertension, diabetes mellitus, coronary heart disease, old myocardial infarction, chronic obstructive pulmonary disease, valvular disease, ejection fraction, smoking, alcohol use, HH diameter, HH volume/thorax volume (HHV/TV), cardiothoracic ratio, and TPVVD were all taken into account. A receiver operating characteristic (ROC) curve was plotted to evaluate the diagnostic accuracy of the identified independent predictors of AF recurrence.

## 3. Results

### 3.1. Patient and clinical characteristics

Table 1 represents patient and clinical characteristics. One hundred eighty people were recruited in this trial: 126 had HH alone, and 54 had HH with AF. The patients’ average age was 72.6 ± 11.1 years. The ratio of male to female was 0.8. Hypertension, hyperlipidemia, diabetes mellitus, coronary heart disease, old myocardial infarction, chronic obstructive pulmonary disease, increased creatinine, and valvular disease were present in, respectively, 52.2%, 30.6%, 15.6%, 37.8%, 2.8%, 13.9%, 11.1%, and 71.7% of patients. History of smoking and of alcohol use were in 20.6% and 13.3%, respectively. Intriguingly, we discovered that AF occurred in 12.7% of patients with hyperlipidemia and 37.6% of patients with normal blood lipid levels (*P* = .001), suggesting that hyperlipidemia is inversely associated with the development of AF in patients with HH.

**Table 1:** Baseline characteristics and radiologic variables.

Variables	All patients (n = 180)	HH + AF (n = 54)	HH (n = 126)	*P*-value
*Baseline characteristics*				
Male/female	80/100	19/35	61/65	.102
Age (years)	72.61 ± 11.1	77.26 ± 8.27	70.81 ± 11.48	**.001**
Hypertension, n (%)	94 (52.2)	34 (62.9)	60 (47.6)	.059
Hyperlipidemia, n (%)	55 (30.6)	7 (13)	48 (38.1)	**.001**
DM, n (%)	28 (15.6)	9 (16.7)	19 (15.1)	.788
CHD, n (%)	68 (37.8)	30 (55.6)	38 (30.1)	**.001**
OMI, n (%)	5 (2.8)	0 (0)	5 (4)	.324
COPD, n (%)	25 (13.9)	8 (14.8)	17 (13.5)	.814
Increased creatinine, n (%)	20 (11.1)	9 (16.7)	11 (8.7)	.121
EF	64.99 ± 5.35	63.65 ± 7.11	65.57 ± 4.3	.17
Valvular disease, n (%)	129 (71.7)	45 (83.3)	84 (66.7)	**.023**
Smoking, n (%)	37 (20.6)	7 (13)	30 (10.3)	.099
Alcohol use, n (%)	24 (13.3)	4 (7.4)	20 (15.9)	.155
*Radiologic parameters*				
RSVD (mm)	15.52 ± 1.92	14.51 ± 2.02	15.95 ± 1.70	**<.001**
RSHD (mm)	13.89 ± 1.75	13.52 ± 1.97	14.05 ± 1.63	.053
RIVD (mm)	14.74 ± 1.81	13.88 ± 2.21	15.11 ± 1.48	**<.001**
RIHD (mm)	12.79 ± 1.83	12.66 ± 2.19	12.85 ± 1.66	.232
LSVD (mm)	15.55 ± 1.84	14.81 ± 2.18	15.86 ± 1.58	**<.001**
LSHD (mm)	14.03 ± 1.82	13.97 ± 2.22	14.06 ± 1.62	.303
LIVD (mm)	15.08 ± 1.94	14.61 ± 2.63	15.28 ± 1.52	**<.001**
LIHD (mm)	13.67 ± 1.80	13.94 ± 2.11	13.54 ± 1.64	.612
TPVVD (mm)	60.89 ± 5.94	57.81 ± 7.34	62.21 ± 4.68	**<.001**
HH diameter (mm)	39.19 ± 20.67	40.44 ± 19.93	38.65 ± 21.04	.67
HHV/TV	0.357 ± 0.057	0.028 ± 0.03	0.498 ± 0.849	.505
Cardiothoracic ratio (%)	49.45 ± 7.19	54.30 ± 7.24	47.37 ± 6.1	**<.001**

AF = atrial fibrillation, CHD = coronary heart disease, COPD = chronic obstructive pulmonary disease, DM = diabetes mellitus, EF = ejection fraction, HH = hiatal hernia, HHV = hiatal hernia volume, LIHD = left inferior horizontal diameter, LIVD = left inferior vertical diameter, LSHD = left superior horizontal diameter, LSVD = left superior vertical diameter, OMI = obsolete myocardial infarction, RIHD = right inferior horizontal diameter, RIVD = right inferior vertical diameter, RSHD = right superior horizontal diameter, RSVD = right superior vertical diameter, TPVVD = total pulmonary vein vertical diameter, TV = thorax volume.

Bolded data are statistically significant.

### 3.2. Radiologic parameters and analysis of patients

In our study population, patients with AF have thinner right superior vertical diameter (14.51 ± 2.02 vs 15.95 ± 1.7 mm), right inferior vertical diameter (13.88 ± 2.21 vs 15.11 ± 1.48 mm), left superior vertical diameter (14.81 ± 2.18 vs 15.86 ± 1.58 mm), left inferior vertical diameter (14.61 ± 2.63 vs 15.28 ± 1.52 mm), and TPVVD (57.81 ± 7.34 vs 62.21 ± 4.68 mm) than those HH alone. There was a statistically significant difference in the cardiothoracic ratio between the 2 groups (0.54 ± 0.07 vs 0.475 ± 0.061). Other radiographic data, such as HHV/TV and HH diameter, were similar in both the groups. Although there was no statistically significant difference in HHV/TV between the 2 groups, the results showed that no individual in the AF group had an HHV/TV >0.01, whereas 20% of individuals in the HH group had an HHV/TV >0.01. This parameter is statistically significant in the *χ*^2^ test analysis (*P* = .026), however, it showed a modest correlation (contingency coefficient of 0.13).

### 3.3. Correlation analysis of TPVVD and cardiothoracic ratio with HH concurrent AF

**Figure 2.**
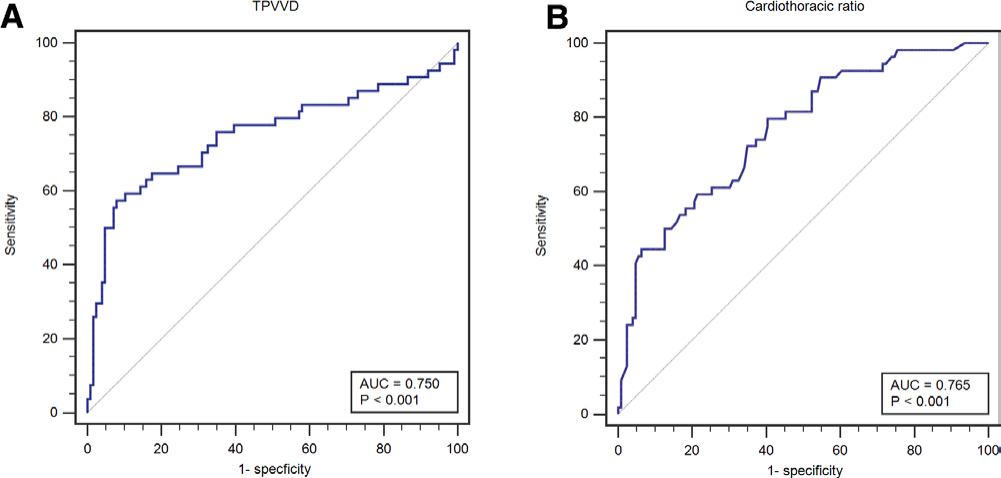
ROC curve plotted for the TPVVD and cardiothoracic ratio. ROC, receiver operating characteristic.

Logistic multivariate regression analysis demonstrated that TPVVD (OR 0.86, 95% confidence interval [CI] 0.80–0.93) and cardiothoracic ratio (OR 1.14, 95% CI 1.06–1.22) were independent predictors of the development of AF with HH (Table 2). Figure 2A and B showed the ROC curve of the TPVVD and cardiothoracic ratio. The area under the ROC curve AUC, indicating the diagnostic accuracy of the TPVVD and cardiothoracic ratio, were calculated as 0.754 (95% CI: 0.68–0.819, sensitivity 57.41%, specificity 91.35%) and 0.765 (95% CI: 0.696–0.825, sensitivity 79.63%, specificity 59.52%), respectively.

**Table 2:** Variables examined in the multivariate regression analysis.

Variables	AF (Model 1 univariate analysis)		AF (Model 2 multivariate analysis)	
	OR (95% CI)	*P*-value	OR (95% CI)	*P*-value
TPVVD	0.86 (0.80–0.92)	**<.01**	0.86 (0.80–0.93)	**<.01**
Cardiothoracic ratio	1.16 (1.10–1.23)	**<.01**	1.14 (1.06–1.22)	**<.01**
Age	1.07 (1.03–1.11)	**<.01**	0.99 (0.95–1.05)	.97
Hypertension	3.52 (1.81–6.86)	**<.01**	2.38 (0.98–5.78)	.06
Valvular disease	3.36 (1.40–8.06)	**<.01**	2.23 (0.77–7.06)	.13
Hyperlipidemia	0.24 (0.10–0.58)	**<.01**	0.16 (0.05–0.47)	**<.01**
CHD	2.90 (1.50–5.60)	**<.01**	2.63 (1.09–6.28)	**.03**

CI = confidence interval, OD = odds ratio. Model 1: unadjusted. Model 2: adjusted age, hypertension, valvular disease, CHD.

Bolded data are statistically significant.

## 4. Discussion

Cases of HH combined with AF have been reported in recent years; however, the definitive relationship between them is unknown. In this investigation, we found statistically significant differences in TPVVD and cardiothoracic ratio between the HH and HH + AF groups, and multivariate correlation analysis revealed that TPVVD and cardiothoracic ratio were independent risk factors for HH leading to AF.

Epidemiological studies have revealed that the prevalence of AF in Asian populations is about 1%, which is less than the prevalence of 2% in Whites.^[2]^ The prevalence of AF among individuals in this study was 5.4%, which is similar to that reported in a previous large-scale study.^[9]^ We evaluated common risk factors for AF in the HH and HH + AF groups and observed no statistically significant differences except for age and valvular disease (Table 1). This finding to imply that the clinical features of AF in patients with HH differ from those of AF in the general population. A major study spanning 50 years demonstrates that advanced age is an independent risk factor for AF.^[16]^ In our study, individuals with HH complicated by AF were older than those without AF, which is consistent with the result of prior investigations.^[9]^ However, the multivariate regression analysis showed that age and valvular disease had no significant correlation with HH complicated AF, indicating that they did not play a major role in HH-related AF (Table 2).

We were confused by the observation that multifactorial regression analysis showed that hyperlipidemia was negatively associated with the development of AF (OR 0.213, 95% CI 0.077–0.594). However, our findings are consistent with those of a recent large-scale clinical trial conducted in Sweden.^[17]^ Several recent studies have discovered that blood lipid levels in northern Chinese individuals gradually decrease around the age of 75.^[18–20]^ In our investigation, the HH + AF group was found to be 77.26 ± 8.27 years, while the HH group was 70.81 ± 11.48 years. This may explains why lipid levels are inversely related to the occurrence of AF.

Previous studies have indicated that patients with AF have larger pulmonary veins than non-AF patients, although the exact mechanism is unclear.^[21]^ Hypertension is an independent risk factor for AF.^[22]^ Hypertension leads to increased pressure in the left atrium, resulting in structural remodeling during migration of the pulmonary veins from the atria and thickening of the ostial pulmonary veins, which in turn is more likely to trigger AF.^[23]^ In animal models, increased intra-atrial pressure increases the rate and organization of waves emanating from the superior pulmonary veins underlying stretch-related AF.^[24]^ According to the studies mentioned above, 1 of the mechanisms by which hypertension causes AF is probably an increase in pressure, which results in pulmonary vein thickening and remodeling. Surprisingly, contrary to prior research, we observed that HH patients with thinner pulmonary veins were more likely to develop AF. We hypothesize that this is caused by HH protruding into the thoracic cavity, which results in a higher venous return resistance. Similar to the increased pressure in the pulmonary veins caused by hypertension, finer pulmonary vein walls are under more pressure.^[25]^ In this study, the volume of HH was not statistically different between the 2 groups, which may indicate that the diameter of the pulmonary vein, rather than the volume of HH, is the key cause of HH complicated by AF. It has also been reported that laparoscopic repair of a large esophageal hiatal hernia compressing the left atrium can prevent the onset of paroxysmal postprandial AF.^[12]^ This may be a test of our suspicions. We also discovered that patients with HH with a higher cardiothoracic ratio were more likely to experience AF complications. A higher cardiothoracic ratio indicated a smaller chest volume, and HH was more likely to result in increased pulmonary venous pressure (Table 1). This may indicate that the pathogenesis of AF induced by HH differs from that in the general population. To our knowledge, this is the first study to investigate the relationship between AF and pulmonary vein diameter in patients with HH.

This study has the following limitations: first, it is a retrospective, single-center study, and a prospective, multi-center study with a larger sample size is required to confirm our conclusions. In addition, not every patient was contrasted with a contrast medium for pulmonary vein visualization, despite the fact that we used two-person repeated measures and compared imaging data to those of previous studies and found no statistically significant differences. Furthermore, some data, such as risk factors for AF, BMI, and chronic renal insufficiency, were not included due to incomplete clinical information.

## 5. Conclusion

This study offers valuable insights into the relationship between TPVVD, cardiothoracic ratio, and the co-occurrence of HH with AF. Contrary to the typical pathogenesis of AF, it is observed that patients with HH have a smaller TPVVD, and an increased cardiothoracic ratio is associated with a higher likelihood of developing AF. The findings indicate that TPVVD and cardiothoracic ratio may serve as independent risk factors for AF in individuals with HH, contributing to a better understanding of the unique pathogenetic characteristics of this rare complication. However, further prospective studies are required to validate these associations and explore the underlying mechanisms involved.

## Acknowledgments

We thank Xuekui Liu (Xuzhou Central Hospital, Xuzhou School of Clinical Medicine of Nangjing Medical University) for his technical assistance.

## Author contributions

**Conceptualization:** Bowen Xu, Xueshan Zhang, Tao Chen, Hongping Chen.

**Data curation:** Xueshan Zhang, Ran Zhou.

**Formal analysis:** Wei Qian.

**Investigation:** Tao Chen.

**Supervision:** Yanfeng Ma.

**Writing – original draft:** Bowen Xu.

**Writing – review & editing:** Yanfeng Ma, Hongping Chen.
